# Supplementary material for: HIVprotI: an integrated web based platform for prediction and design of HIV proteins inhibitors
Source: J Cheminform. 2018 Mar 9;10:12. doi: 10.1186/s13321-018-0266-y (PMC5845081; doi:10.1186/s13321-018-0266-y)
Supplement: Supplementary file 2 — Additional file 2. Source code of HIVProtI web server. [file 13321_2018_266_MOESM2_ESM.zip › HIVprotI_Source-code/hivprotI/weka-3-6-6/doc/weka/classifiers/trees/m5/RuleNode.html]

RuleNode


---


|  |  |  |
| --- | --- | --- |
| **Overview** \| **Package** \| **Class** \| **Tree** \| **Deprecated** \| **Index** \| **Help** \| **Weka's home** | |  |
| **PREV CLASS**   **NEXT CLASS** | **FRAMES**    **NO FRAMES**     **All Classes** |  |
| SUMMARY: NESTED \| FIELD \| CONSTR \| METHOD | DETAIL: FIELD \| CONSTR \| METHOD |  |


---


## weka.classifiers.trees.m5 Class RuleNode

```
java.lang.Object
  weka.classifiers.Classifier
      weka.classifiers.trees.m5.RuleNode
```

**All Implemented Interfaces:**: java.io.Serializable, java.lang.Cloneable, CapabilitiesHandler, OptionHandler, RevisionHandler

---

``` public class RuleNode extends Classifier ```

Constructs a node for use in an m5 tree or rule

**Version:**
:   $Revision: 1.13 $

**Author:**
:   Mark Hall (mhall@cs.waikato.ac.nz)

**See Also:**: Serialized Form

---

| **Field Summary** | |
| --- | --- |
| `int` | `m_numParameters`             the number of paramters in the chosen model for this node---either the subtree model or the linear model. |


**Constructor Summary**

`RuleNode(double globalDev,
double globalAbsDev,
RuleNode parent)`
  
          Creates a new `RuleNode` instance.


| **Method Summary** | |
| --- | --- |
| `void` | `buildClassifier(Instances data)`             Build this node (find an attribute and split point) |
| `double` | `classifyInstance(Instance inst)`             Classify an instance using this node. |
| `void` | `findBestLeaf(double[] maxCoverage, RuleNode[] bestLeaf)`             Find the leaf with greatest coverage |
| `double` | `getMinNumInstances()`             Get the minimum number of instances to allow at a leaf node |
| `PreConstructedLinearModel` | `getModel()`             Get the linear model at this node |
| `int` | `getNumInstances()`             Return the number of instances that reach this node. |
| `boolean` | `getRegressionTree()`             Get the value of regressionTree. |
| `java.lang.String` | `getRevision()`             Returns the revision string. |
| `void` | `graph(java.lang.StringBuffer text)`             Assign a unique identifier to each node in the tree and then calls graphTree |
| `void` | `installLinearModels()`             Traverses the tree and installs linear models at each node. |
| `void` | `installSmoothedModels()` |
| `boolean` | `isLeaf()`             Return true if this node is a leaf |
| `RuleNode` | `leftNode()`             Get the left child of this node |
| `java.lang.String` | `nodeToString()`             Returns a description of this node (debugging purposes) |
| `int` | `numberOfLinearModels()`             Get the number of linear models in the tree |
| `int` | `numLeaves(int leafCounter)`             Sets the leaves' numbers |
| `RuleNode` | `parentNode()`             Get the parent of this node |
| `void` | `printAllModels()`             Print all the linear models at the learf (debugging purposes) |
| `java.lang.String` | `printLeafModels()`             print all leaf models |
| `java.lang.String` | `printNodeLinearModel()`             print the linear model at this node |
| `void` | `prune()`             Recursively prune the tree |
| `void` | `returnLeaves(FastVector[] v)`             Return a list containing all the leaves in the tree |
| `RuleNode` | `rightNode()`             Get the right child of this node |
| `void` | `setMinNumInstances(double minNum)`             Set the minumum number of instances to allow at a leaf node |
| `void` | `setRegressionTree(boolean newregressionTree)`             Set the value of regressionTree. |
| `void` | `split()`             Finds an attribute and split point for this node |
| `int` | `splitAtt()`             Get the index of the splitting attribute for this node |
| `double` | `splitVal()`             Get the split point for this node |
| `java.lang.String` | `toString()`             print the linear model at this node |
| `java.lang.String` | `treeToString(int level)`             Recursively builds a textual description of the tree |

**Methods inherited from class weka.classifiers.Classifier**

`debugTipText, distributionForInstance, forName, getCapabilities, getDebug, getOptions, listOptions, makeCopies, makeCopy, setDebug, setOptions`

**Methods inherited from class java.lang.Object**

`equals, getClass, hashCode, notify, notifyAll, wait, wait, wait`

**Field Detail**

### m\_numParameters

```
public int m_numParameters
```

:   the number of paramters in the chosen model for this node---either
    the subtree model or the linear model.
    The constant term is counted as a paramter---this is for pruning
    purposes


**Constructor Detail**

### RuleNode

```
public RuleNode(double globalDev,
                double globalAbsDev,
                RuleNode parent)
```

:   Creates a new `RuleNode` instance.

    **Parameters:**: `globalDev` - the global standard deviation of the class: `globalAbsDev` - the global absolute deviation of the class: `parent` - the parent of this node


**Method Detail**

### buildClassifier

```
public void buildClassifier(Instances data)
                     throws java.lang.Exception
```

:   Build this node (find an attribute and split point)

    :   **Specified by:**: `buildClassifier` in class `Classifier`
    :   **Parameters:**: `data` - the instances on which to build this node **Throws:**: `java.lang.Exception` - if an error occurs

---


### classifyInstance

```
public double classifyInstance(Instance inst)
                        throws java.lang.Exception
```

:   Classify an instance using this node. Recursively calls classifyInstance
    on child nodes.

    :   **Overrides:**: `classifyInstance` in class `Classifier`
    :   **Parameters:**: `inst` - the instance to classify **Returns:**: the prediction for this instance **Throws:**: `java.lang.Exception` - if an error occurs

---


### split

```
public void split()
           throws java.lang.Exception
```

:   Finds an attribute and split point for this node

    :   **Throws:**: `java.lang.Exception` - if an error occurs

---


### numLeaves

```
public int numLeaves(int leafCounter)
```

:   Sets the leaves' numbers

    :   **Parameters:**: `leafCounter` - the number of leaves counted **Returns:**: the number of the total leaves under the node

---


### toString

```
public java.lang.String toString()
```

:   print the linear model at this node

    :   **Overrides:**: `toString` in class `java.lang.Object`
    :   **Returns:**: the linear model

---


### printNodeLinearModel

```
public java.lang.String printNodeLinearModel()
```

:   print the linear model at this node

    :   **Returns:**: the linear model at this node

---


### printLeafModels

```
public java.lang.String printLeafModels()
```

:   print all leaf models

    :   **Returns:**: the leaf models

---


### nodeToString

```
public java.lang.String nodeToString()
```

:   Returns a description of this node (debugging purposes)

    :   **Returns:**: a string describing this node

---


### treeToString

```
public java.lang.String treeToString(int level)
```

:   Recursively builds a textual description of the tree

    :   **Parameters:**: `level` - the level of this node **Returns:**: string describing the tree

---


### installLinearModels

```
public void installLinearModels()
                         throws java.lang.Exception
```

:   Traverses the tree and installs linear models at each node.
    This method must be called if pruning is not to be performed.

    :   **Throws:**: `java.lang.Exception` - if an error occurs

---


### installSmoothedModels

```
public void installSmoothedModels()
                           throws java.lang.Exception
```

:   **Throws:**: `java.lang.Exception`

---


### prune

```
public void prune()
           throws java.lang.Exception
```

:   Recursively prune the tree

    :   **Throws:**: `java.lang.Exception` - if an error occurs

---


### findBestLeaf

```
public void findBestLeaf(double[] maxCoverage,
                         RuleNode[] bestLeaf)
```

:   Find the leaf with greatest coverage

    :   **Parameters:**: `maxCoverage` - the greatest coverage found so far: `bestLeaf` - the leaf with the greatest coverage

---


### returnLeaves

```
public void returnLeaves(FastVector[] v)
```

:   Return a list containing all the leaves in the tree

    :   **Parameters:**: `v` - a single element array containing a vector of leaves

---


### parentNode

```
public RuleNode parentNode()
```

:   Get the parent of this node

    :   **Returns:**: the parent of this node

---


### leftNode

```
public RuleNode leftNode()
```

:   Get the left child of this node

    :   **Returns:**: the left child of this node

---


### rightNode

```
public RuleNode rightNode()
```

:   Get the right child of this node

    :   **Returns:**: the right child of this node

---


### splitAtt

```
public int splitAtt()
```

:   Get the index of the splitting attribute for this node

    :   **Returns:**: the index of the splitting attribute

---


### splitVal

```
public double splitVal()
```

:   Get the split point for this node

    :   **Returns:**: the split point for this node

---


### numberOfLinearModels

```
public int numberOfLinearModels()
```

:   Get the number of linear models in the tree

    :   **Returns:**: the number of linear models

---


### isLeaf

```
public boolean isLeaf()
```

:   Return true if this node is a leaf

    :   **Returns:**: true if this node is a leaf

---


### getModel

```
public PreConstructedLinearModel getModel()
```

:   Get the linear model at this node

    :   **Returns:**: the linear model at this node

---


### getNumInstances

```
public int getNumInstances()
```

:   Return the number of instances that reach this node.

    :   **Returns:**: the number of instances at this node.

---


### getRegressionTree

```
public boolean getRegressionTree()
```

:   Get the value of regressionTree.

    :   **Returns:**: Value of regressionTree.

---


### setMinNumInstances

```
public void setMinNumInstances(double minNum)
```

:   Set the minumum number of instances to allow at a leaf node

    :   **Parameters:**: `minNum` - the minimum number of instances

---


### getMinNumInstances

```
public double getMinNumInstances()
```

:   Get the minimum number of instances to allow at a leaf node

    :   **Returns:**: a `double` value

---


### setRegressionTree

```
public void setRegressionTree(boolean newregressionTree)
```

:   Set the value of regressionTree.

    :   **Parameters:**: `newregressionTree` - Value to assign to regressionTree.

---


### printAllModels

```
public void printAllModels()
```

:   Print all the linear models at the learf (debugging purposes)

---


### graph

```
public void graph(java.lang.StringBuffer text)
```

:   Assign a unique identifier to each node in the tree and then
    calls graphTree

    :   **Parameters:**: `text` - a `StringBuffer` value

---


### getRevision

```
public java.lang.String getRevision()
```

:   Returns the revision string.

    :   **Specified by:**: `getRevision` in interface `RevisionHandler` **Overrides:**: `getRevision` in class `Classifier`
    :   **Returns:**: the revision


---


|  |  |  |
| --- | --- | --- |
| **Overview** \| **Package** \| **Class** \| **Tree** \| **Deprecated** \| **Index** \| **Help** \| **Weka's home** | |  |
| **PREV CLASS**   **NEXT CLASS** | **FRAMES**    **NO FRAMES**     **All Classes** |  |
| SUMMARY: NESTED \| FIELD \| CONSTR \| METHOD | DETAIL: FIELD \| CONSTR \| METHOD |  |


---
